# Supplementary material for: The clinicopathological significance of SWI/SNF alterations in gastric cancer is associated with the molecular subtypes
Source: PLoS One. 2021 Jan 22;16(1):e0245356. doi: 10.1371/journal.pone.0245356 (PMC7822341; doi:10.1371/journal.pone.0245356)
Supplement: S1 Table — (DOC) [file pone.0245356.s001.doc]

**S1 Table.** Univariate and multivariate analysis of prognostic factors in patients with gastric cancer according to overall survival.

| Factors | Mean (months) | 95% C.I. | *P* value | Hazard ratio | 95% C.I. | *P* value |
| --- | --- | --- | --- | --- | --- | --- |
| Age |  |  | 0.129 |  |  | 0.007 |
| ≤65 (n=594) | 123.16 | 113.98-132.33 |  | 1 |  |  |
| >65 (n=629) | 111.30 | 102.46-120.14 |  | 1.268 | 1.067-1.506 |  |
| Gender |  |  | 0.206 |  |  |  |
| Male (n=772) | 115.58 | 107.29-123.87 |  |  |  |  |
| Female (n=451) | 123.25 | 112.87-133.63 |  |  |  |  |
| Gastrectomy |  |  | <0.001 |  |  | <0.001 |
| Proximal/subtotal (n=846) | 134.15 | 126.38-141.93 |  | 1 |  |  |
| Total (n=377) | 82.50 | 71.57-93.43 |  | 1.700 | 1.263-2.289 |  |
| Lymphadenectomy |  |  | 0.043 |  |  | <0.001 |
| D1/D1+ (n=300) | 108.48 | 95.15-121.80 |  | 1 |  |  |
| D2 (n=923) | 120.74 | 113.41-128.07 |  | 0.670 | 0.548-0.820 |  |
| Stump Cancer |  |  | 0.002 |  |  | 0.032 |
| No (n=1164) | 121.44 | 114.73-128.16 |  | 1 |  |  |
| Yes (n=59) | 65.93 | 45.09-86.77 |  | 1.605 | 1.041-2.475 |  |
| Location |  |  | <0.001 |  |  | 0.005 |
| Upper (n=212) | 104.75 | 89.54-119.97 |  | 0.583 | 0.416-0.818 | 0.002 |
| Middle (n=223) | 122.07 | 107.03-137.12 |  | 0.631 | 0.468-0.851 | 0.003 |
| Lower (n=742) | 126.06 | 117.73-134.40 |  | 1 |  |  |
| Diffuse (n=46) | 18.04 | 12.78-23.30 |  | 0.645 | 0.405-1.028 | 0.065 |
| Tumor size (cm) |  |  | <0.001 |  |  | 0.003 |
| ≤5 (n=773) | 145.01 | 136.96-153.07 |  | 1 |  |  |
| >5 (n=450) | 73.09 | 63.51-82.68 |  | 1.316 | 1.096-1.580 |  |
| Differentiation |  |  | <0.001 |  |  | 0.590 |
| WD/MD (n=464) | 141.46 | 130.98-151.94 |  | 1 |  |  |
| PD (n=759) | 103.89 | 95.86-111.93 |  | 1.071 | 0.835-1.372 |  |
| Combined classification |  |  | <0.001 |  |  | <0.001 |
| MSI (n=114) | 149.20 | 131.10-167.30 |  | 0.438 | 0.301-0.637 | <0.001 |
| EBV (n=65) | 137.16 | 109.03-165.28 |  | 0.400 | 0.254-0.629 | <0.001 |
| Intestinal (n=466) | 130.46 | 120.11-140.82 |  | 0.749 | 0.621-0.905 | 0.003 |
| Diffuse/Mixed (n=530) | 89.52 | 80.18-98.87 |  | 1 |  |  |
| Stage |  |  | <0.001 |  |  | <0.001 |
| I (n=274) | 208.63 | 200.58-216.68 |  | 1 |  |  |
| II (n=246) | 164.79 | 151.49-178.09 |  | 3.075 | 1.961-4.824 | <0.001 |
| III (n=588) | 68.08 | 60.33-75.82 |  | 9.692 | 6.423-14.624 | <0.001 |
| IV(n=115) | 14.92 | 9.86-19.97 |  | 21.273 | 13.128-34.471 | <0.001 |
| Resection margins |  |  | <0.001 |  |  | 0.017 |
| Negative (n=1089) | 128.21 | 121.32-135.11 |  | 1 |  |  |
| Positive (n=134) | 38.39 | 25.71-51.07 |  | 1.356 | 1.055-1.743 |  |
| Lymphatic invasion^a^ |  |  | <0.001 |  |  | 0.135 |
| No (n=512) | 173.48 | 164.47-182.49 |  | 1 |  |  |
| Yes (n=690) | 75.94 | 68.15-83.74 |  | 1.194 | 0.946-1.507 |  |
| Vascular invasion^a^ |  |  | <0.001 |  |  | 0.020 |
| No (n=1007) | 132.78 | 125.58-139.98 |  | 1 |  |  |
| Yes (n=188) | 51.00 | 38.88-63.12 |  | 1.274 | 1.039-1.564 |  |
| Perineural invasion^a^ |  |  | <0.001 |  |  | 0.343 |
| No (n=562) | 158.35 | 149.49-167.21 |  | 1 |  |  |
| Yes (n=634) | 81.45 | 73.03-89.87 |  | 1.101 | 0.902-1.344 |  |
| HER2 status^a^ |  |  | 0.235 |  |  |  |
| Negative (n=853) | 125.71 | 118.05-133.36 |  |  |  |  |
| Positive (n=59) | 123.60 | 99.87-147.33 |  |  |  |  |
| SWI/SNF status |  |  | 0.149 |  |  | 0.001 |
| Retained | 121.48 | 113.43-129.54 |  | 1 |  |  |
| Attenuated | 113.74 | 102.78-124.71 |  | 1.360 | 1.138-1.625 |  |

EBV, Epstein-Barr virus; MSI, microsatellite instability; LN ratio, ratio of metastatic to retrieved lymph nodes ; C.I., confidence interval

^a^ Not all data were available
