# Supplementary material for: The clinicopathological significance of SWI/SNF alterations in gastric cancer is associated with the molecular subtypes
Source: PLoS One. 2021 Jan 22;16(1):e0245356. doi: 10.1371/journal.pone.0245356 (PMC7822341; doi:10.1371/journal.pone.0245356)
Supplement: S2 Table — (DOC) [file pone.0245356.s002.doc]

**S2 Table.** Univariate and multivariate analysis of prognostic factors in patients with stage II-IV gastric cancer, including chemotherapy data.

| Factors | Median (months) | 95% CI | *P* value | Hazard ratio | 95% CI | *P* value |
| --- | --- | --- | --- | --- | --- | --- |
| Age |  |  | 0.123 |  |  | 0.028 |
| ≤65 (n=465) | 31.5 | 24.93-38.08 |  | 1 |  |  |
| >65 (n=484) | 24.5 | 19.75-29.25 |  | 1.259 | 1.025-1.546 |  |
| Gender |  |  | 0.517 |  |  |  |
| Female (n=345) | 28.2 | 21.73-34.67 |  |  |  |  |
| Male (n=604) | 28.0 | 23.63-32.37 |  |  |  |  |
| Gastrectomy |  |  | <0.001 |  |  | 0.350 |
| Proximal/subtotal (n=609) | 33.2 | 27.65-38.75 |  | 1 |  |  |
| Total (n=340) | 18.2 | 15.43-20.97 |  | 1.110 | 0.892-1.382 |  |
| Lymphadenectomy |  |  | 0.004 |  |  | 0.006 |
| D1/D1+ (n=218) | 22.4 | 14.49-30.31 |  | 1 |  |  |
| D2 (n=731) | 28.8 | 24.48-33.12 |  | 0.702 | 0.545-0.903 |  |
| Stump Cancer |  |  | 0.034 |  |  | 0.324 |
| No (n=898) | 28.6 | 24.83-32.37 |  | 1 |  |  |
| Yes (n=51) | 13.7 | 8.83-18.57 |  | 1.389 | 0.723-2.670 |  |
| Location |  |  | <0.001 |  |  | 0.239 |
| Upper (n=186) | 30.7 | 19.23-42.17 |  | 0.670 | 0.438-1.025 | 0.065 |
| Middle (n=170) | 26.1 | 12.18-40.02 |  | 0.753 | 0.531-1.068 | 0.112 |
| Lower (n=547) | 30.4 | 25.98-34.82 |  | 1 |  |  |
| Diffuse (n=46) | 12.0 | 8.29-15.71 |  | 0.841 | 0.462-1.531 | 0.571 |
| Tumor size (cm) |  |  | <0.001 |  |  | 0.008 |
| ≤5 (n=520) | 41.4 | 32.79-49.81 |  | 1 |  |  |
| >5 (n=429) | 18.9 | 16.24-21.56 |  | 1.327 | 1.076-1.637 |  |
| Differentiation |  |  | <0.001 |  |  | 0.701 |
| WD/MD (n=317) | 37.8 | 26.22-49.38 |  | 1 |  |  |
| PD (n=632) | 23.7 | 20.10-27.30 |  | 1.062 | 0.781-1.445 |  |
| Combined classification |  |  | <0.001 |  |  | <0.001 |
| EBV (n=54) | 46.1 | 0-125.95 |  | 0.546 | 0.332-0.899 | 0.017 |
| MSI (n=84) | - | - |  | 0.485 | 0.320-0.735 | 0.001 |
| Intestinal (n=339) | 34.7 | 24.01-45.39 |  | 0.690 | 0.550-0.866 | 0.001 |
| Diffuse/Mixed (n=462) | 19.9 | 17.15-22.65 |  | 1 |  |  |
| Stage |  |  | <0.001 |  |  | <0.001 |
| II (n=246) | - | - |  | 1 |  |  |
| III (n=588) | 22.4 | 19.49-25.31 |  | 3.109 | 2.342-4.126 | <0.001 |
| IV(n=115) | 8.9 | 6.14-11.66 |  | 6.246 | 3.129-12.468 | <0.001 |
| Resection margins |  |  | <0.001 |  |  | 0.023 |
| Negative (n=816) | 32.3 | 27.02-37.59 |  | 1 |  |  |
| Positive (n=133) | 14.1 | 10.79-17.41 |  | 1.421 | 1.048-1.925 |  |
| Lymphatic invasion^a^ |  |  | <0.001 |  |  | 0.314 |
| No (n=264) | 144.0 | - |  | 1 |  |  |
| Yes (n=669) | 20.7 | 17.92-23.48 |  | 1.155 | 0.873-1.527 |  |
| Vascular invasion^a^ |  |  | <0.001 |  |  | 0.001 |
| No (n=740) | 33.2 | 27.01-39.39 |  | 1 |  |  |
| Yes (n=186) | 15.8 | 11.89-19.71 |  | 1.500 | 1.175-1.914 |  |
| Perineural invasion^a^ |  |  | <0.001 |  |  | 0.983 |
| No (n=313) | 48.2 | 14.66-81.75 |  | 1 |  |  |
| Yes (n=611) | 23.4 | 19.78-27.02 |  | 1.003 | 0.793-1.267 |  |
| HER2 status^a^ |  |  | 0.047 |  |  | 0.127 |
| Negative (n=633) | 30.9 | 25.42-36.38 |  | 1 |  |  |
| Positive (n=49) | 63.3 | - |  | 0.720 | 0.472-1.098 |  |
| SWI/SNF status |  |  | 0.565 |  |  | 0.019 |
| Retained (n=602) | 29.0 | 23.79-34.22 |  | 1 |  |  |
| Attenuated (n=347) | 25.3 | 20.20-30.40 |  | 1.291 | 1.043-1.597 |  |
| Chemotherapy |  |  | <0.001 |  |  | <0.001 |
| Negative (n=243) | 15.8 | 11.85-19.75 |  | 1 |  |  |
| Positive (n=703) | 32.0 | 26.71-37.29 |  | 0.595 | 0.462-0.766 |  |

WD/MD, well differentiated/moderately differentiated; PD, poorly differentiated; EBV, Epstein-Barr virus; MSI, microsatellite instability; NA, not available; CI, confidence interval

^a^, Not all data were available
