# Supplementary material for: The clinicopathological significance of SWI/SNF alterations in gastric cancer is associated with the molecular subtypes
Source: PLoS One. 2021 Jan 22;16(1):e0245356. doi: 10.1371/journal.pone.0245356 (PMC7822341; doi:10.1371/journal.pone.0245356)
Supplement: S3 Table — (DOCX) [file pone.0245356.s003.docx]

**S3 Table.** Clinicopathological differences between SMARCA2-retained and SMARCA2-attenuated gastric cancers.

| Parameters | Total  (n = 1224) | SMARCA2- lost  (n = 131) | SMARCA2- reduced  (n = 143) | SMARCA2- heterogeneous  (n = 70) | SMARCA2  -retained  (n =880) | *P* value |
| --- | --- | --- | --- | --- | --- | --- |
| Age (median ± SD, yrs) | 66.00 ± 13.65 | 66.00 ± 12.99 | 68.00 ± 13.65 | 67.00 ± 12.80 | 66.00 ± 13.90 | 0.362 |
| ≤ 65 | 594 (48.5) | 62 (47.3) | 65 (45.5) | 28 (40) | 439 (49.9) | 0.347 |
| > 65 | 630 (51.5) | 69 (52.7) | 78 (54.5) | 42 (60) | 441 (50.1) |  |
| Sex |  |  |  |  |  | 0.055 |
| Male | 773 (63.2) | 81 (61.8) | 99 (69.2) | 35 (50) | 558 (63.4) |  |
| Female | 451 (36.8) | 50 (38.2) | 44 (30.8) | 35 (50) | 322 (36.6) |  |
| Gastrectomy |  |  |  |  |  | 0.021 |
| Proximal/Subtotal | 847 (69.2) | 80 (61.1) | 95 (66.4) | 42 (60) | 630 (71.6) |  |
| Total | 377 (30.8) | 51 (38.9) | 48 (33.6) | 28 (40) | 250 (28.4) |  |
| Lymphadenectomy |  |  |  |  |  | 0.533 |
| D1/D1+ | 301 (24.6) | 35 (26.7) | 32 (22.4) | 13 (18.6) | 221 (25.1) |  |
| D2 | 923 (75.4) | 96 (73.3) | 111 (77.6) | 57 (81.4) | 659 (74.9) |  |
| Stump cancer |  |  |  |  |  | 0.167 |
| Yes | 59 (4.8) | 11 (8.4) | 8 (5.6) | 4 (5.7) | 36 (4.1) |  |
| No | 1165 (95.2) | 120 (91.6) | 135 (94.4) | 66 (94.3) | 844 (72.4) |  |
| Localization |  |  |  |  |  | 0.514 |
| Upper | 212 (17.3) | 29 (22.1) | 143 (16.3) | 13 (18.6) | 27 (18.9) |  |
| Middle | 223 (18.2) | 26 (19.8) | 152 (17.3) | 12 (17.1) | 33 (23.1) |  |
| Lower | 743 (60.7) | 70 (53.4) | 552 (62.7) | 42 (60.0) | 79 (55.2) |  |
| Diffuse | 46 (3.8) | 6 (4.6) | 33 (3.8) | 3 (4.3) | 4 (2.8) |  |
| Size (median ± SD, cm) | 4.0 ± 3.60 | 5.0 ± 3.43 | 4.3 ± 2.96 | 4.5 ± 3.81 | 4.0 ± 3.71 | 0.387 |
| ≤ 5 | 774 (63.2) | 74 (56.5) | 90 (62.9) | 46 (65.7) | 564 (64.1) |  |
| > 5 | 450 (36.8) | 57 (43.5) | 53 (37.1) | 24 (34.3) | 316 (35.9) |  |
| Differentiation |  |  |  |  |  | 0.005 |
| WD/MD | 465 (38.0) | 31 (23.7) | 56 (39.2) | 29 (41.4) | 349 (39.7) |  |
| PD | 759 (62.0) | 100 (76.3) | 87 (60.8) | 41 (58.6) | 531 (60.3) |  |
| Lauren’s classification |  |  |  |  |  | 0.031 |
| Intestinal | 592 (48.4) | 48 (36.6) | 76 (53.1) | 35 (50) | 433 (49.2) |  |
| Diffuse/Mixed | 632 (51.6) | 83 (63.4) | 67 (46.9) | 35 (50) | 447 (50.8) |  |
| Genotypes^a^ |  |  |  |  |  | <0.001 |
| EBV | 65 (5.5) | 22 (17.1) | 16 (11.9) | 3 (4.7) | 24 (2.8) |  |
| MSI | 114 (9.7) | 9 (7.0) | 13 (9.7) | 8 (7.0) | 84 (9.9) |  |
| Intestinal | 467 (39.7) | 31 (24.0) | 55 (41.0) | 29 (45.3) | 352 (41.5) |  |
| Diffuse/Mixed | 530 (45.1) | 67 (51.9) | 50 (37.3) | 24 (37.5) | 389 (45.8) |  |
| Depth of invasion |  |  |  |  |  | 0.062 |
| T1 | 202 (16.5) | 18 (13.7) | 16 (11.2) | 13 (18.6) | 155 (17.6) |  |
| T2 | 161 (13.2) | 20 (15.3) | 17 (11.9) | 13 (18.6) | 111 (12.6) |  |
| T3 | 280 (22.9) | 19 (14.5) | 42 (29.4) | 13 (18.6) | 206 (23.4) |  |
| T4 | 581 (47.5) | 74 (56.5) | 68 (47.6) | 31 (44.3) | 408 (46.4) |  |
| Nodal status |  |  |  |  |  | 0.099 |
| N0 | 414 (33.8) | 41 (31.3) | 36 (25.2) | 20 (28.6) | 317 (36.0) |  |
| N1 | 158 (12.9) | 17 (13.0) | 16 (11.2) | 10 (14.3) | 115 (13.1) |  |
| N2 | 208 (17.0) | 16 (12.2) | 27 (18.9) | 15 (21.4) | 150 (17.0) |  |
| N3 | 444 (36.3) | 57 (43.5) | 64 (44.8) | 25 (35.7) | 298 (33.9) |  |
| LN ratio, median ± SD | 0.14 ± 0.30 | 0.18 ± 0.32 | 0.18 ± 0.31 | 0.11 ± 0.30 | 0.13 ± 0.29 | 0.034 |
| Distant metastasis |  |  |  |  |  | 0.526 |
| M0 | 1109 (90.6) | 115 (87.8) | 133 (93) | 63 (90) | 798 (90.7) |  |
| M1 | 115 (9.4) | 16 (12.2) | 10 (7) | 7 (10) | 82 (9.3) |  |
| Stage |  |  |  |  |  | 0.183 |
| I | 275 (22.5) | 28 (21.4) | 21 (14.7) | 18 (25.7) | 208 (23.6) |  |
| II | 246 (20.1) | 21 (16.0) | 28 (19.6) | 13 (18.6) | 184 (20.9) |  |
| III | 588 (48.0) | 66 (50.4) | 84 (58.7) | 32 (45.7) | 406 (46.1) |  |
| IV | 115 (9.4) | 16 (12.2) | 10 (7.0) | 7 (10.0) | 82 (9.3) |  |
| Resection margins |  |  |  |  |  | 0.485 |
| Negative | 1090 (89.1) | 115 (87.8) | 124 (86.7) | 60 (85.7) | 791 (89.9) |  |
| Positive | 134 (10.9) | 16 (12.2) | 19 (13.3) | 10 (14.3) | 89 (10.1) |  |
| Lymphatic invasion^a^ |  |  |  |  |  | <0.001 |
| No | 513 (42.6) | 46 (35.9) | 39 (27.3) | 31 (44.9) | 397 (46.0) |  |
| Yes | 690 (57.4) | 82 (64.1) | 104 (72.7) | 38 (55.1) | 466 (54.0) |  |
| Vascular invasion^a^ |  |  |  |  |  | 0.952 |
| No | 1008 (84.3) | 108 (85.7) | 118 (83.7) | 59 (85.5) | 723 (84.1) |  |
| Yes | 188 (15.7) | 18 (14.3) | 23 (16.3) | 10 (14.5) | 137 (15.9) |  |
| Perineural invasion^a^ |  |  |  |  |  | 0.711 |
| No | 563 (47.0) | 61 (48.4) | 61 (42.7) | 34 (49.3) | 407 (47.4) |  |
| Yes | 634 (53.0) | 65 (51.6) | 82 (57.3) | 35 (50.7) | 452 (52.6) |  |
| HER2 status^a^ |  |  |  |  |  | 0.876 |
| Negative | 853 (93.5) | 84 (93.3) | 101 (95.3) | 49 (94.2) | 619 (93.2) |  |
| Positive | 59 (6.5) | 6 (6.7) | 5 (4.7) | 3 (5.8) | 45 (6.8) |  |
| Locoregional recurrence^b^ |  |  |  |  |  | 0.251 |
| Negative | 354 (67.9) | 38 (76) | 47 (66.2) | 24 (80) | 245 (66.2) |  |
| Positive | 167 (32.1) | 12 (24) | 24 (33.8) | 6 (20) | 125 (33.8) |  |
| Peritoneal recurrence^b^ |  |  |  |  |  | 0.914 |
| Negative | 322 (61.8) | 29 (58) | 43 (60.6) | 18 (60) | 232 (62.7) |  |
| Positive | 199 (38.2) | 21 (42) | 28 (39.4) | 12 (40) | 138 (37.3) |  |
| Hematogenous recurrence^b^ |  |  |  |  |  | 0.800 |
| Negative | 343 (65.8) | 33 (66.0) | 48 (67.6) | 22 (73.3) | 240 (64.9) |  |
| Positive | 178 (34.2) | 17 (34.0) | 23 (32.4) | 8 (26.7) | 130 (35.1) |  |
| Lymph node recurrence^b^ |  |  |  |  |  | 0.854 |
| Negative | 414 (79.5) | 40 (80) | 56 (78.9) | 22 (73.3) | 296 (80) |  |
| Positive | 107 (20.5) | 10 (20) | 15 (21.1) | 8 (26.7) | 74 (20) |  |
| Chemotherapy^c^ |  |  |  |  |  | 0.007 |
| Negative | 243 (25.7) | 39 (37.9) | 28 (23.0) | 18 (34.6) | 158 (23.6) |  |
| Positive | 703 (74.3) | 64 (62.1) | 94 (77.0) | 34 (65.4) | 511 (76.4) |  |

Figures are numbers with percentages in parentheses.

EBV, Epstein-Barr virus; MSI, microsatellite instable; SD, standard deviation; WD/MD, well differentiated/moderately differentiated; PD, poorly differentiated; LN ratio, ratio of metastatic to retrieved lymph nodes.

^a^ Not all data were available

^b^ Stage I-III cases with available data regarding recurrence site.

^c^ Stage II-IV cases with available data of chemotherapy
