# Supplementary material for: The clinicopathological significance of SWI/SNF alterations in gastric cancer is associated with the molecular subtypes
Source: PLoS One. 2021 Jan 22;16(1):e0245356. doi: 10.1371/journal.pone.0245356 (PMC7822341; doi:10.1371/journal.pone.0245356)
Supplement: S4 Table — (DOCX) [file pone.0245356.s004.docx]

**S4 Table.** Clinicopathological differences between ARID1A-retained and ARID1A-attenuated gastric cancers.

| Parameters | Total  (n = 1224) | ARID1A- lost  (n = 125) | ARID1A - reduced  (n = 20) | ARID1A - heterogeneous  (n = 52) | ARID1A  -retained  (n =1027) | *P* value |
| --- | --- | --- | --- | --- | --- | --- |
| Age (median ± SD, yrs) | 66.00 ± 13.65 | 69.00 ± 13.23 | 63.00 ± 10.52 | 66.50 ± 14.14 | 66.00 ± 13.72 | 0.230 |
| ≤ 65 | 594 (48.5) | 53 (42.4) | 12 (60) | 24 (46.2) | 505 (49.2) | 0.359 |
| > 65 | 630 (51.5) | 72 (57.6) | 8 (40) | 28 (53.8) | 522 (50.8) |  |
| Sex |  |  |  |  |  | 0.567 |
| Male | 773 (63.2) | 73 (58.4) | 14 (70) | 35 (67.3) | 651 (63.4) |  |
| Female | 451 (36.8) | 52 (41.6) | 6 (30) | 17 (32.7) | 376 (36.6) |  |
| Gastrectomy |  |  |  |  |  | 0.128 |
| Proximal/Subtotal | 847 (69.2) | 85 (68) | 11 (55) | 30 (57.7) | 721 (70.2) |  |
| Total | 377 (30.8) | 40 (32) | 9 (45) | 22 (42.3) | 306 (29.8) |  |
| Lymphadenectomy |  |  |  |  |  | 0.193 |
| D1/D1+ | 301 (24.6) | 32 (25.6) | 9 (45) | 13 (25) | 247 (24.1) |  |
| D2 | 923 (75.4) | 93 (74.4) | 11 (55) | 39 (75) | 780 (75.9) |  |
| Stump cancer |  |  |  |  |  | <0.001 |
| Yes | 59 (4.8) | 119 (95.2) | 14 (70) | 47 (90.4) | 985 (95.9) |  |
| No | 1165 (95.2) | 6 (4.8) | 6 (30) | 5 (9.6) | 42 (4.1) |  |
| Localization |  |  |  |  |  | 0.089 |
| Upper | 212 (17.3) | 23 (18.4) | 3 (15) | 13 (25.0) | 173 (16.8) |  |
| Middle | 223 (18.2) | 26 (20.8) | 9 (45) | 10 (19.2) | 178 (17.3) |  |
| Lower | 743 (60.7) | 73 (58.4) | 7 (35) | 28 (53.8) | 635 (61.8) |  |
| Diffuse | 46 (3.8) | 3 (2.4) | 1 (5) | 1 (1.9) | 3 (2.4) |  |
| Size (median ± SD, cm) | 4.0 ± 3.60 | 5.0 ± 3.28 | 4.25 ± 2.65 | 4.25 ± 2.97 | 4.0 ± 3.69 | 0.222 |
| ≤ 5 | 774 (63.2) | 67 (53.6) | 14 (70) | 33 (63.5) | 660 (64.3) | 0.119 |
| > 5 | 450 (36.8) | 58 (46.4) | 6 (30) | 19 (36.5) | 367 (35.7) |  |
| Differentiation |  |  |  |  |  | 0.166 |
| WD/MD | 465 (38.0) | 38 (30.4) | 5 (25) | 20 (38.5) | 402 (39.1) |  |
| PD | 759 (62.0) | 87 (69.6) | 15 (75) | 32 (61.5) | 625 (60.9) |  |
| Lauren’s classification |  |  |  |  |  | 0.257 |
| Intestinal | 592 (48.4) | 66 (52.8) | 6 (30) | 23 (44.2) | 497 (48.4) |  |
| Diffuse/Mixed | 632 (51.6) | 59 (47.2) | 14 (70) | 29 (55.8) | 530 (51.6) |  |
| Genotypes^a^ |  |  |  |  |  | <0.001 |
| EBV | 65 (5.5) | 19 (15.8) | 7 (38.9) | 4 (8.0) | 35 (3.5) |  |
| MSI | 114 (9.7) | 40 (33.3) | 2 (11.1) | 11 (22.0) | 61 (6.2) |  |
| Intestinal | 467 (39.7) | 27 (22.5) | 3 (16.7) | 16 (32.0) | 421 (42.6) |  |
| Diffuse/Mixed | 530 (45.1) | 34 (28.3) | 6 (33.3) | 19 (38.0) | 471 (47.7) |  |
| Depth of invasion |  |  |  |  |  | 0.234 |
| T1 | 202 (16.5) | 12 (9.6) | 1 (5) | 9 (17.3) | 180 (17.5) |  |
| T2 | 161 (13.2) | 17 (13.6) | 2 (10) | 10 (19.2) | 132 (12.9) |  |
| T3 | 280 (22.9) | 33 (26.4) | 8 (40) | 11 (21.2) | 228 (22.2) |  |
| T4 | 581 (47.5) | 63 (50.4) | 9 (45) | 22 (42.3) | 487 (47.4) |  |
| Nodal status |  |  |  |  |  | 0.374 |
| N0 | 414 (33.8) | 43 (34.4) | 8 (40) | 22 (42.3) | 341 (33.2) |  |
| N1 | 158 (12.9) | 22 (17.6) | 2 (10) | 6 (11.5) | 128 (12.5) |  |
| N2 | 208 (17.0) | 13 (10.4) | 2 (10) | 6 (11.5) | 187 (18.2) |  |
| N3 | 444 (36.3) | 47 (37.6) | 8 (40) | 18 (34.6) | 371 (36.1) |  |
| LN ratio, median ± SD | 0.14 ± 0.30 | 0.11 ± 0.27 | 0.11 ± 0.35 | 0.11 ± 0.33 | 0.14 ± 0.30 | 0.796 |
| Distant metastasis |  |  |  |  |  | 0.794 |
| M0 | 1109 (90.6) | 115 (92.0) | 17 (85) | 47 (90.4) | 930 (90.6) |  |
| M1 | 115 (9.4) | 10 (8.0) | 3 (15) | 5 (9.6) | 97 (9.4) |  |
| Stage |  |  |  |  |  | 0.046 |
| I | 275 (22.5) | 21 (16.8) | 1 (5) | 16 (30.8) | 237 (23.1) |  |
| II | 246 (20.1) | 36 (28.8) | 7 (35) | 12 (23.1) | 191 (18.6) |  |
| III | 588 (48.0) | 58 (46.4) | 9 (45) | 19 (36.5) | 502 (48.9) |  |
| IV | 115 (9.4) | 10 (8.0) | 3 (15) | 5 (9.6) | 97 (7.9) |  |
| Resection margins |  |  |  |  |  | 0.681 |
| Negative | 1090 (89.1) | 108 (86.4) | 18 (90) | 48 (92.3) | 916 (89.2) |  |
| Positive | 134 (10.9) | 17 (13.6) | 2 (10) | 4 (7.7) | 111 (10.8) |  |
| Lymphatic invasion^a^ |  |  |  |  |  | 0.361 |
| No | 513 (42.6) | 50 (40.7) | 5 (25) | 24 (47.1) | 434 (43.0) |  |
| Yes | 690 (57.4) | 73 (59.3) | 15 (75) | 27 (52.9) | 575 (57.0) |  |
| Vascular invasion^a^ |  |  |  |  |  | 0.462 |
| No | 1008 (84.3) | 105 (87.5) | 14 (73.7) | 42 (84.0) | 847 (84.1) |  |
| Yes | 188 (15.7) | 15 (12.5) | 5 (26.3) | 8 (16.0) | 160 (15.9) |  |
| Perineural invasion^a^ |  |  |  |  |  | 0.966 |
| No | 563 (47.0) | 58 (47.2) | 10 (50) | 24 (48.0) | 471 (46.9) |  |
| Yes | 634 (53.0) | 65 (52.8) | 9 (45) | 26 (52.0) | 534 (53.1) |  |
| HER2 status^a^ |  |  |  |  |  | 0.038 |
| Negative | 853 (93.5) | 96 (100) | 11 (100) | 30 (93.8) | 716 (92.6) |  |
| Positive | 59 (6.5) | 0 (0) | 0 (0) | 2 (6.3) | 57 (7.4) |  |
| Locoregional recurrence^b^ |  |  |  |  |  | 0.775 |
| Negative | 354 (67.9) | 34 (69.4) | 7 (77.8) | 13 (76.5) | 300 (67.3) |  |
| Positive | 167 (32.1) | 15 (30.6) | 2 (22.2) | 4 (23.5) | 146 (32.7) |  |
| Peritoneal recurrence^b^ |  |  |  |  |  | 0.508 |
| Negative | 322 (61.8) | 27 (55.1) | 4 (44.4) | 11 (64.7) | 280 (62.8) |  |
| Positive | 199 (38.2) | 22 (44.9) | 5 (55.6) | 6 (35.3) | 166 (37.2) |  |
| Hematogenous recurrence^b^ |  |  |  |  |  | 0.131 |
| Negative | 343 (65.8) | 35 (71.4) | 7 (77.8) | 15 (88.2) | 286 (64.1) |  |
| Positive | 178 (34.2) | 14 (28.6) | 2 (22.2) | 2 (11.8) | 160 (35.9) |  |
| Lymph node recurrence^b^ |  |  |  |  |  | 0.201 |
| Negative | 414 (79.5) | 39 (79.6) | 7 (77.8) | 10 (58.8) | 358 (80.3) |  |
| Positive | 107 (20.5) | 10 (20.4) | 2 (22.2) | 7 (41.2) | 88 (19.7) |  |
| Chemotherapy^c^ |  |  |  |  |  | 0.145 |
| Negative | 243 (25.7) | 35 (33.7) | 5 (26.3) | 12 (33.3) | 191 (24.3) |  |
| Positive | 703 (74.3) | 69 (66.3) | 14 (73.7) | 24 (66.7) | 596 (75.7) |  |

Figures are numbers with percentages in parentheses.

EBV, Epstein-Barr virus; MSI, microsatellite instable; SD, standard deviation; WD/MD, well differentiated/moderately differentiated; PD, poorly differentiated; LN ratio, ratio of metastatic to retrieved lymph nodes.

^a^ Not all data were available

^b^ Stage I-III cases with available data regarding recurrence site.

^c^ Stage II-IV cases with available data of chemotherapy
